# Post‐Banding Ulcer Bleeding After Endoscopic Ligation: Incidence, Risk Factors and Outcomes in Patients With Cirrhosis

**DOI:** 10.1111/apt.70495

**Published:** 2025-12-10

**Authors:** Maria de Brito Nunes, Matthias Knecht, Jonas Schropp, Reiner Wiest, Jaume Bosch, Annalisa Berzigotti

**Affiliations:** ^1^ Department of Visceral Surgery and Medicine, Inselspital, Bern University Hospital University of Bern Bern Switzerland; ^2^ Department of Internal Medicine Hospital of Fribourg Fribourg Switzerland; ^3^ Graduate School for Health Sciences (GHS) University of Bern Bern Switzerland

**Keywords:** endoscopic band ligation, liver cirrhosis, oesophageal and gastric varices, post‐banding ulcer bleeding

## Abstract

**Background and Study Aim:**

Post‐banding ulcer bleeding (PBUB) is an understudied complication of endoscopic band ligation (EBL) used in the prevention and treatment of oesophageal variceal bleeding (VB). The aim of this study is to investigate the incidence, mortality and risk factors of PBUB.

**Methods:**

Retrospective cohort study conducted at the university hospital of Bern (Switzerland). It included patients with cirrhosis and oesophageal varices who underwent prophylactic or urgent EBL for VB between 1 January 2018 and 31 December 2022.

**Results:**

In total, 206 patients with cirrhosis, who underwent 630 sessions of EBL, were included. The incidence rate of PBUB was 17.5% (95% CI, 12.7%–23.5%), considering the total number of patients, and 6.8% (95% CI, 5.0%–9.2%) considering the total of EBL procedures. Urgent EBL (SHR: 2.78, 95% CI: 1.29–6.00, *p* = 0.009) and elevated creatinine (SHR: 1.04, 95% CI: 1.01–1.07, *p* = 0.024) were independent risk factors for PBUB on multivariate analysis. PBUB required blood product transfusions in 88.1% of events (95% CI, 73.6%–95.5%) and hospitalisation at the intensive care unit in 74.4% of events, with a median hospital stay of 2 days (range: 1–34 days). In patients with PBUB, the short‐term mortality during hospitalisation was 19%, and long‐term mortality at 52 weeks was 64%.

**Conclusions:**

Patients with cirrhosis undergoing urgent EBL or those with elevated creatinine levels are at increased risk of PBUB. Due to the high mortality associated with PBUB, identifying high‐risk patients and implementing preventive strategies is essential for improving patient outcomes.

## Graphical abstract

A PBUB is a rare but often fatal complication of endoscopic variceal band ligation. The relevant risk factors contributing to PBUB are unknown and consequently there are no effective preemptive strategies. This study highlights kidney disease as a predictor of PBUB together with the well‐established higher risk for urgent band ligation.

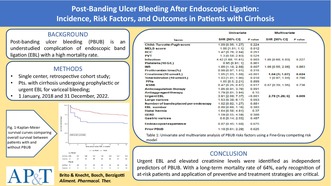

**Abbreviations**
ACLF: acute‐on‐chronic liver failure
ALD: alcohol‐associated liver disease
CI: confidence interval
CRP: C‐reactive protein
EBL: endoscopic band ligation
GERD: gastro‐oesophageal reflux disease
HCC: hepatocellular carcinoma
HR: hazard ratio
INR: international normalised ratio
MASLD: metabolic dysfunction‐associated liver disease
MELD score: model for end‐stage liver disease score
NSBB: non‐selective beta blockers
OLT: orthotopic liver transplantation
PBU: post‐banding ulcers
PBUB: post‐banding ulcer bleeding
PPIs: proton pump inhibitors
PVT: portal vein thrombosis
SHR: sub‐distribution hazard ratio
TIPS: transjugular intrahepatic portosystemic shunt
VB: oesophageal variceal bleeding

## 1 Introduction

Oesophageal variceal bleeding (VB) is a major concern in patients with liver disease. About 60%–65% of patients with cirrhosis and portal hypertension will develop VB before death or liver transplantation [1]. Despite optimal management of decompensated liver disease, mortality remains high, ranging from 10% to 20% [2, 3, 4], and is closely associated with the degree of hepatic dysfunction [5, 6].

In contemporary clinical practice, endoscopic band ligation (EBL) is one of the mainstay treatments of VB both in the prevention of rebleeding as well as in primary prophylaxis in case of contraindication or intolerance to non‐selective beta blockers (NSBB) [7].

Thought EBL is efficacious in treating VB, it can lead to several complications. Among those, post‐banding ulcer bleeding (PBUB) is of particular concern. PBUB arises from ulcers that develop at sites where ligation bands were applied. Its pathophysiology is, however, still poorly understood. A recent systematic review of our group reported an occurrence of PBUB in 5.5% of EBL, with a mortality rate of 22.5% due to this complication [8].

This study investigated the incidence, mortality and risk factors of PBUB in a tertiary referral hospital, with the aim of expanding and validating the results of the above‐mentioned systematic review.

## 2 Methods

### 2.1 Study Design and Participants

We conducted a monocentric retrospective cohort study at the University Hospital of Bern (Switzerland). Our study included data on EBL procedures conducted for primary prophylaxis, secondary prophylaxis, or emergency treatment in patients with cirrhosis and portal hypertension, from 1 January 2018 to 31 December 2022.

The study population included patients with cirrhosis and portal hypertension with oesophageal varices, who underwent EBL. Cirrhosis was diagnosed using clinical criteria, ultrasonographic findings, and in a part of the cases, by liver biopsy. There was no upper limit of age for participants, MELD score, or Child‐Pugh class. Patients with congenital bleeding disorders or those who refused to provide the general informed consent for the use of their clinical data were excluded.

### 2.2 Procedures

The EBL procedure was performed using a standard adult endoscope (Olympus GIF HQ190; Olympus Medical Systems, Hamburg, Germany) by either a senior endoscopist or a senior gastroenterology resident. The bands were applied using a 6 ShooterSaeed Multi‐Band Ligator MBL‐6 (Cook Medical LLC, Indiana, USA).

Following the initial index EBL, additional endoscopic procedures were performed as necessary at 2–6 weeks intervals, until the complete eradication of varices was achieved. A follow‐up endoscopy was done at least 3 months after eradication. The indication for EBL was categorised as either urgent EBL in instances where patients were admitted within the first 24 h due to acute VB, or prophylactic for non‐emergency EBL, including both primary and secondary prophylaxis. In accordance with the American Association for the Study of Liver Diseases (AASLD) guidelines [9], varices were classified as either small or large, with or without red signs or white nipple signs. PBUB was ascertained as the source of upper gastrointestinal bleeding after EBL, if one or more ulcers were observed at the ligation site, without any other identifiable potential source of bleeding. The diagnosis of PBUB was established by an experienced endoscopist. PBUB was defined according to the endoscopic appearance using the Jamwal & Sarin classification [10] of PBUB: Ulcer with spurting (type A); Ulcer with oozing (type B); Ulcer with clot or pigmented base (type C); Ulcer with clean base (type D). Representative examples of these ulcer types are illustrated in Table 1.

**TABLE 1:** Classification of post‐banding ulcers (PBUs) according to Jamwal et al. [10].

Type	Description	
A	Ulcer with spurting	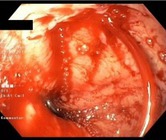
B	Ulcer with oozing	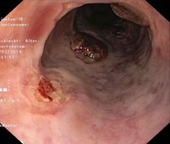
C	Ulcer with clot or pigmented base	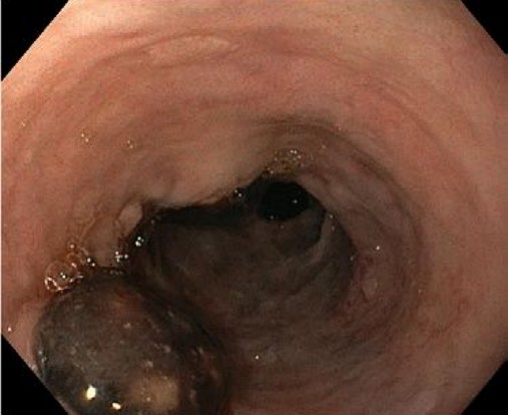
D	Ulcer with clean base	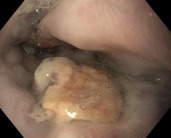

*Note:* Adapted from Jamwal et al. [10].

### 2.3 Data Collection and Variables

The patients with cirrhosis treated with EBL were identified by one of the investigators in collaboration with the *lnsel* Data Science Centre using the endoscopy database from the gastroenterology department. An independent investigator reviewed all electronic patient files for inclusion criteria and constructed a new database during 2024 with anonymised data on RedCap electronic data capture tools hosted at University of Bern. To ensure the integrity and precision of the gathered information, endoscopic findings in patients presenting PBUB were reviewed by an investigator experienced in endoscopy. The senior investigator reviewed the final database.

The study involved an examination of multiple variables, including demographic, clinical, medication, laboratory and endoscopic procedure parameters collected all at index EBL, subsequent endoscopies with EBL, and at follow‐up assessments (Table S1). These procedure parameters were: setting of EBL, grade and site of treated varices, number of bands placed per endoscopy and per varix, presence of high‐risk stigmata such as red wale sign, nipple sign, cherry red spots and fibrin plug, presence of hiatal hernia and its assessment using the Hill classification, endoscopic evidence of gastro‐oesophageal reflux disease (GERD), and experience of the endoscopist.

Data from patients was collected from the index EBL and all subsequent EBLs until their final scheduled endoscopic assessment, loss to follow‐up, transplantation, or death.

This study is reported according to the *Strengthening the Reporting of Observational Studies in Epidemiology* (STROBE) statement.

### 2.4 Study Outcomes

The primary outcomes were the overall incidence of bleeding due to post‐banding ulcers (PBU) and risk factors of PBUB. Secondary outcomes included the occurrence of PBU, complications after endoscopy (pain, dysphagia, fever, vomiting and weight loss), blood products transfused, stay in the intensive care unit (ICU), hospitalisation length, management of PBUB, short‐term and long‐term mortality, survival rates and causes of death.

Short‐term mortality was assessed at three time points: in‐hospital mortality, defined as death occurring during the same hospitalisation in which EBL was performed; 28‐day mortality, defined as death occurring within 28 days of the index EBL; 90‐day mortality, defined as death occurring within 90 days of the index EBL. Long‐term mortality was defined as death occurring at 52 weeks (365 days) or more after the index EBL.

### 2.5 Ethics

The study was done according to the Declaration of Helsinki and Good Clinical Practice guidelines. Included participants or their legal relatives signed a written informed consent of the University Hospital of Bern. The study was approved by the Ethics Committee of Canton Bern (Nr: 2022‐00941).

### 2.6 Statistical Analysis

Continuous variables were summarised as median and interquartile range [Q1–Q3], and frequency and percentage for categorical variables. *p*‐values are provided for the baseline table based on whether a patient would later experience PBUB at least once, using the Wilcoxon rank sum test for continuous variables and either Pearson's Chi‐squared test or Fisher's exact test for categorical variables, depending on expected cell counts.

The cumulative incidence of PBUB was visualised for relevant covariates. Associations were assessed using univariate Fine–Grey competing risks sub‐distribution hazards models for each potential risk factor. Cluster‐robust standard errors were used to account for repeated EBL procedures within the same patient. Significant risk factors were then included in a multivariate model.

OS (overall survival) and the time until PBUB were assessed and visualised from the time of EBL using the Kaplan–Meier (KM) method. Survival probabilities are provided at 7, 14, 28, 90 and 365 days after index EBL. Event rates based on the KM estimator are given for PBUB and orthotopic liver transplantation (OLT), survival rates for OS. The Fine‐Grey sub‐distribution hazards model was used to estimate the cumulative incidence function for PBUB, accounting for the competing event of OLT and death. Due to clustering within patients, confidence intervals were estimated using the infinitesimal jackknife variance estimate. To assess the association of PBUB with OS, it was included as a time‐varying covariate.

Statistical analysis was conducted using R software, version 4.3.2.

## 3 Results

**FIGURE 1:**
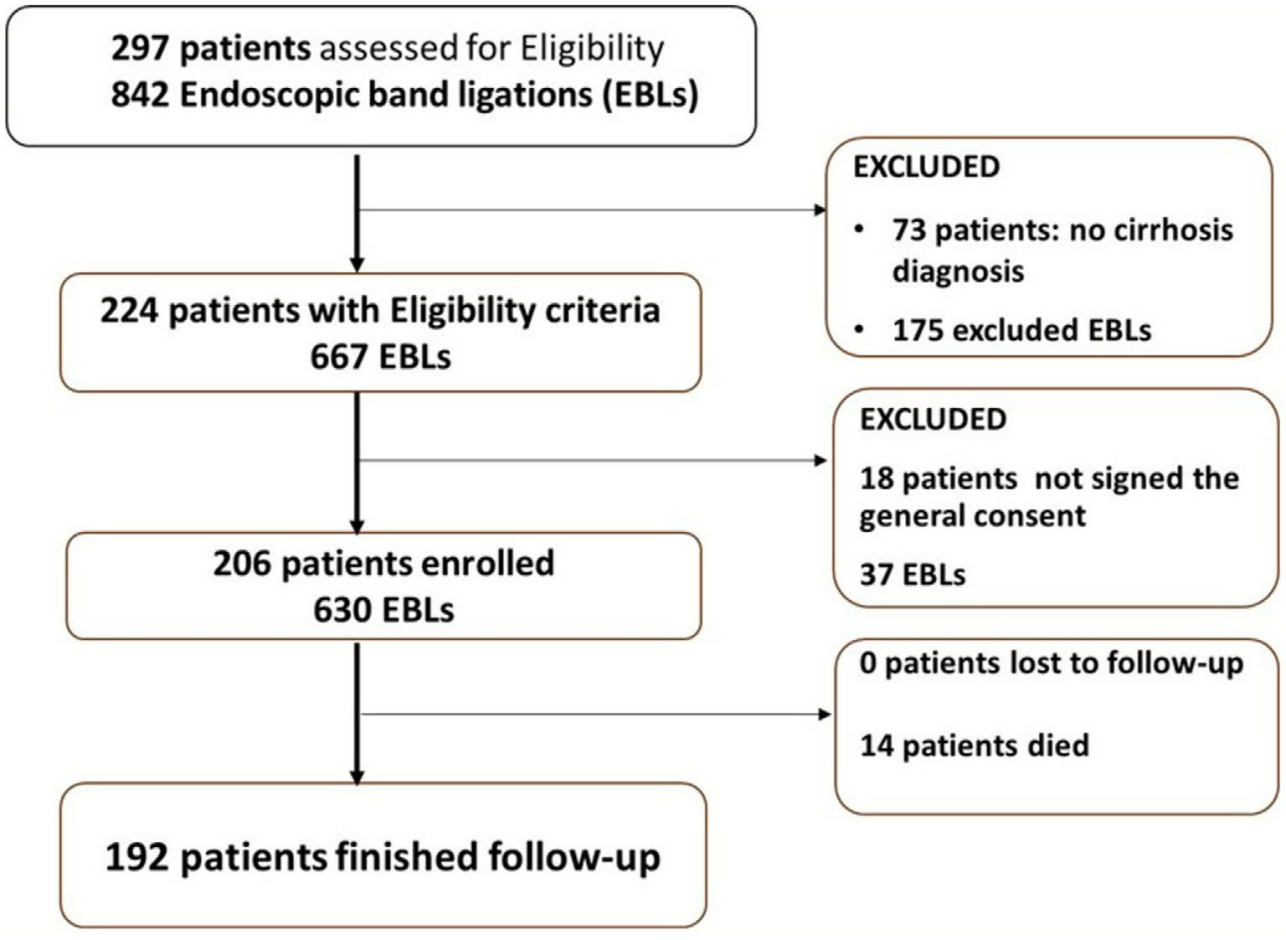
Flowchart detailing patient inclusion and follow‐up.

Our cohort included 206 patients with cirrhosis who underwent 630 sessions of endoscopic band ligation (EBL) at the University Hospital of Bern (Switzerland) between 1 January 2018 and 31 December 2022. Initially, 297 patients were evaluated for eligibility. Of these, 73 patients were excluded because they did not meet the inclusion criteria, and 18 patients declined to provide informed consent for study participation (Figure 1). There were no losses to follow‐up during the study period.

### 3.1 Patient Characteristics

The mean age of the cohort was 62 years, with a predominance of male patients (*n* = 156, 76%). Alcohol‐associated liver disease (ALD) was the most common cause of cirrhosis, affecting 126 patients (61%), followed by metabolic dysfunction‐associated liver disease (MASLD) in 60 patients (29%) and viral hepatitis in 37 patients (18%). Hepatocellular carcinoma (HCC) was present in 56 patients (27%) and portal vein thrombosis (PVT) in 38 patients (23%).

New cirrhosis decompensation was the most common indication for EBL, primarily manifested as upper gastrointestinal bleeding (131 patients, 64%). The etiologies of decompensation included alcohol‐related liver injury in 57 patients (28%), infection in 14 patients (6.8%), a new diagnosis of HCC in 17 patients (8.3%), a new diagnosis of PVT in 10 patients (4.9%) and treatment withdrawal in 2 patients (1%). Detailed data with baseline characteristics of patients is provided in Table S2.

### 3.2 Post‐Banding Ulcer Bleeding After EBL

We observed 43 events of PBUB in 36 patients. The incidence rate of PBUB was 17.5% (95% CI, 12.7%–23.5%), considering the total number of patients, and 6.8% (95% CI, 5.0%–9.2%) considering the number of EBL procedures. When analysed by indication, the incidence of PBUB was higher for urgent EBL (13.1%, 95% CI, 8.8%–18.9%) compared to prophylactic EBL (3.6%, 95% CI, 2.2%–6.0%). PBUB was not more frequent in the subgroup of patients with gastroesophageal varices (10%) than in those without (18%, *p* = 0.5; Table S3). The median time between EBL and PBUB was 12 days (IQR: 6–19). EBL inter‐session intervals were similar between patients with PBUB and without PBUB. The distributions overlapped and most sessions occurred within 2–6 weeks (Figure S1). Blood product transfusions were required in 88.1% of PBUB cases (95% CI, 73.6%–95.5%). PBUB required hospitalisation at the intensive care unit in 32 (74.4%) of PBUB events, with a median length of stay of 2 days (range: 1–34 days).

Post‐banding ulcers (PBU) were frequently observed on follow‐up endoscopies. The incidence rate of PBU was 37.9% (95% CI, 31.3%–44.9%) per patient and 19% (95% CI, 16.1%–22.4%) per EBL procedure.

In patients without PBUB, most ulcers encountered were classified as type D (ulcers with a clean base; 90%). In patients with PBUB, ulcers were predominantly type A (ulcer with spurting, 17%), type B (ulcer with oozing, 2.4%), and type C (clot or pigmented base, 56%; Table S4).

Other complications associated with EBL were rare.

### 3.3 Mortality Associated to PBUB After EBL

*Short‐term mortality*: overall 14 (6.8%) patients died during hospitalisation, including 7 (19%) patients with PBUB and 7 (4.1%) without PBUB.

*Long‐term mortality*: 114 (55%) patients died during the entire follow‐up period, including 23 (64%) patients with PBUB and 91 (54%) patients without PBUB.

**FIGURE 2:**
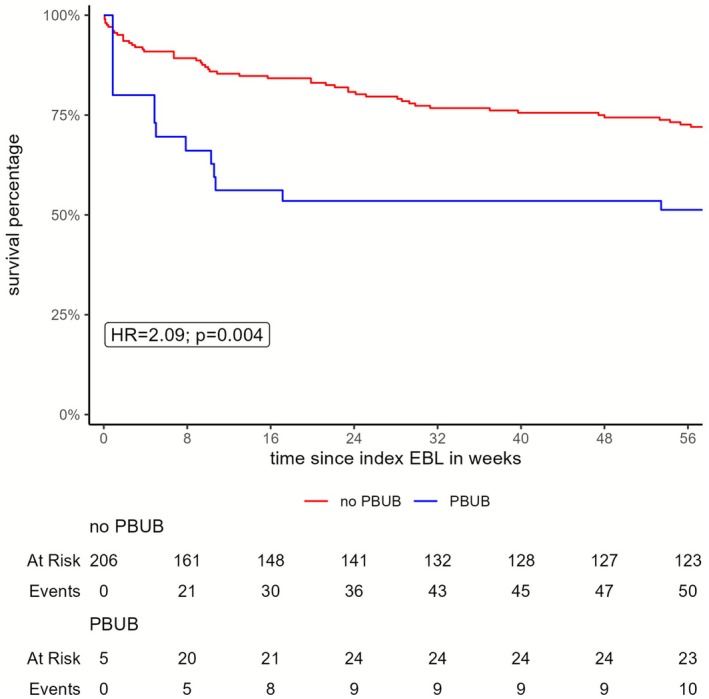
Kaplan–Meier survival curves comparing overall survival between patients with and without PBUB. The log‐rank test indicates a significant difference between the groups (*p* = 0.004). Censored observations are marked with tick marks on the curves.

Overall survival probability was significantly lower in patients with PBUB compared to those without PBUB (HR = 3.86, *p* < 0.001), with the most pronounced decline occurring within 4 months after EBL (Figure 2). At 365 days, survival was 48% (95% CI, 29%–81%) in patients with PBUB and 81% (95% CI, 76%–86%) in those without PBUB.

Subgroup analysis of outpatients undergoing prophylactic EBL shows a short‐term survival at 7 and 14 days of 100% in patients with PBUB as well as without PBUB. Nevertheless, short‐term survival at 28 days and long‐term overall survival at 365 days were lower in patients with PBUB (Table 3). The hazard ratio for PBUB in this subgroup was 8.73 (*p*_Wald = 0.001; robust *p* = 0.10). The attenuated robust *p*‐value reflects the small number of PBUB events.

Patients undergoing urgent EBL had poorer survival than those receiving prophylactic EBL (Table 2). The 365‐day survival in patients receiving prophylactic EBL was 88% (95% CI, 84%–93%) vs 60% (95% CI, 51%–70%) in patients undergoing urgent EBL.

**TABLE 2:** Causes of death among the study cohort, comparing patients with and without PBUB. Some patients had multiple contributing causes of death.

Cause of death	Total patients (*n* = 114)	No PBUB (*n* = 91)	PBUB (*n* = 23)	*p*
Hypovolemic shock due to PBUB	5 (4.4%)	0 (0.0%)	5 (21.7%)	0.001
Hypovolemic shock from other GI origin	17 (14.9%)	14 (15.4%)	3 (13.0%)	0.69
Intra‐cerebral bleeding	2 (1.8%)	2 (2.2%)	0 (0.0%)	1.00
Septic shock	18 (15.8%)	15 (16.5%)	3 (13.0%)	1.00
ACLF	39 (34.2%)	28 (30.8%)	11 (47.8%)	0.18
HCC	25 (21.9%)	20 (22.0%)	5 (21.7%)	1.00
Extra‐hepatic cancers	4 (3.5%)	4 (4.4%)	0 (0.0%)	0.58
Unknown	27 (23.7%)	25 (27.5%)	2 (8.7%)	0.31

Abbreviations: ACLF, acute‐on‐chronic liver failure; HCC, hepatocellular carcinoma; PBUB, post‐banding ulcer bleeding.

**TABLE 3:** Overall survival (OS) of patients with PBUB versus without PBUB, in the whole population and divided by the EBL being urgent or prophylactic EBL, and occurrence in cases of prophylactic EBL.

	OS at 7 days	OS at 14 days	OS at 28 days	OS at 90 days	OS at 365 days
Occurrence of PBUB (HR = 3.86; *p*Wald < 0.001; *p*Lr‐robust < 0.001)					
No PBUB	98% (97%, 99%)	97% (96%, 98%)	95% (93%, 97%)	91% (88%, 94%)	81% (76%, 86%)
PBUB	86% (63%, 100)	86% (63%, 100%)	71% (49%, 100%)	54% (34%, 86%)	48% (29%, 81%)
Indication (HR = 3.82; *p*Wald < 0.001; *p*LR‐robust < 0.001)					
Prophylactic EBL	100%	100%	98% (97%, 100%)	96% (94%, 98%)	88% (84%, 93%)
Urgent EBL	93% (89%, 96%)	90% (86%, 94%)	84% (79%, 89%)	74% (67%, 82%)	60% (51%, 70%)
Occurrence of PBUB in patients with prophylactic EBL (HR = 8.73; *p*Wald = 0.001; *p*Lr‐robust = 0.1)					
No PBUB	100% (100%, 100%)	100% (100%, 100%)	99% (97%, 100%)	97% (95%, 99%)	89% (84%, 93%)
PBUB	100% (100%, 100%)	100% (100%, 100%)	89% (70%, 100%)	57% (29%, 100%)	57% (29%, 100%)

*Note:* Presence of PBUB was included as a time‐varying covariate. OS estimates are presented as percentages with 95% confidence intervals (CIs). CIs were estimated using the infinitesimal jackknife variance estimate due to clustering. Hazard ratio (HR) and *p*‐values were determined using the Cox PH model. pWald = *p*‐value from the Wald test; pLR‐robust = *p*‐value from a robust likelihood‐ratio test (sandwich/Huber–White variance, clustered by patient to account for repeated procedures).

Abbreviations: EBL, endoscopic band ligation; HR, hazard ratio; OS, overall survival; PBUB, post‐banding ulcer bleeding.

**TABLE 4:** Univariate and multivariate analysis of PBUB risk factors using a Fine‐Grey competing risk model.

Term	Univariate		Multivariate	
	SHR [95% CI]	*p*	SHR [95% CI]	*p*
Child–Turcotte–Pugh score	1.09 [0.95; 1.27]	0.224		
MELD score	1.06 [1.01; 1.1]	0.012		
HCC	1.47 [0.76; 2.84]	0.251		
PVT	1.3 [0.58; 2.93]	0.524		
Infection	4.42 [1.68; 11.61]	0.003	1.85 [0.68; 5.03]	0.227
Platelets (10 G/L)	0.95 [0.91; 1]	0.061		
INR	1.85 [1.18; 2.88]	0.007	1.06 [0.55; 2.06]	0.863
Prothrombin time (%)	0.99 [0.97; 1.01]	0.315		
Creatinine (10 umol/L)	1.05 [1.03; 1.08]	< 0.001	**1.04 [1; 1.07]**	**0.024**
Total bilirubin (10 umol/L)	1.03 [1.01; 1.06]	0.018	1 [0.97; 1.04]	0.798
PPIs	1.05 [0.5; 2.21]	0.899		
NSBB	0.47 [0.25; 0.87]	0.017	0.87 [0.39; 1.95]	0.736
Anticoagulation therapy	1.85 [0.91; 3.76]	0.091		
Antiaggregant therapy	1.79 [0.81; 3.94]	0.15		
Urgent EBL	3.91 [2.08; 7.37]	< 0.001	**2.78 [1.29; 6]**	**0.009**
Large varices	1.53 [0.38; 6.17]	0.553		
Number of bands placed per endoscopy	1.02 [0.82; 1.27]	0.881		
EBL number	0.89 [0.68; 1.16]	0.383		
Hiatal hernia	1.64 [0.56; 4.81]	0.37		
GERD	1.59 [0.55; 4.59]	0.388		
Presence of gastro‐oesophageal varices	0.6 [0.14; 2.55]	0.487		
Endoscopist experience: resident, senior doctor	0.87 [0.45; 1.68]	0.675		
Prior PBUB	1.18 [0.61; 2.29]	0.625		

*Note:* Bold values are those statistically significant.

Abbreviations: GERD, gastroesophageal reflux disease; HCC, hepatocellular carcinoma; INR, international normalised ratio; MELD score, model for end‐stage liver disease score; NSBB, non‐selective beta‐blockers; PVT, portal venous thrombosis; PBUB, post‐banding ulcer bleeding; PPIs, proton pump inhibitors; SHR, sub‐distribution hazard ratio.

The causes of death in patients with PBUB were multifactorial (Table 2). Hypovolemic shock due to PBUB occurred in 5 patients, while other causes, shared between both groups, were primarily related to advanced liver failure, severe infections and metastatic HCC.

Liver transplantation was performed in 26 patients (13%) in the full cohort, including 6 patients (17%) with PBUB and 20 patients (12%) without PBUB.

### 3.4 Clinical Outcomes

By the end of the study, patients who developed PBUB had more severe liver failure than those who did not. MELD score was 15 in patients with PBUB versus 11 in patients without PBUB (IQR: PBUB 10–23; No PBUB 9–16, *p* = 0.043). The median Child‐ Turcotte‐Pugh score was worse in patients with PBUB (9, IQR: 8–11) than in patients without PBUB (7, IQR: 6–9, *p* < 0.001).

Decompensation of liver cirrhosis during follow‐up was significantly more frequent in patients with PBUB, with 69% experiencing decompensation in contrast to 27% in patients without PBUB.

At the time of EBL, plasma creatinine was higher in patients who later developed PBUB (median: 91 μmol/L) than in those without PBUB (median: 77 μmol/L; *p* = 0.023). Other parameters, including haemoglobin, platelets, C‐reactive protein (CRP), and international normalised ratio (INR), did not differ significantly between groups (Table S5).

### 3.5 Findings and Treatments Related to EBL

Urgent EBL was performed in 191 occasions (30%), EBL for primary prophylaxis in 136 (22%), and EBL for secondary prophylaxis in 303 (48%). Urgent EBL was more common in patients who developed PBUB, occurring after 25 procedures (61%) opposed to 166 (28%) in patients without PBUB (*p* = 0.001, Table S4). The number needed to ligate to cause one additional PBUB event was 11 urgent EBL procedures. A history of acute VB before inclusion in the study was similar in patients with PBUB (7 patients, 19%) and without PBUB (35 patients, 21%; *p* = 0.9).

The overall mean number of EBL sessions per patient was 3 (SD: 2.33). Patients with PBUB underwent slightly more EBL sessions (mean: 3.56, SD: 2.62) than patients without PBUB (mean: 2.95, SD: 2.26, *p* = 0.14). The median number of bands placed per endoscopy was 3 (IQR: 2.00–4.00) across all procedures and was not related to PBUB incidence (SHR = 0.89 [0.68; 1.16], *p* = 0.881). In patients with PBUB, the median number of bands placed at first endoscopy was 4 (IQR: 2.50–5.00) and 3 (IQR: 2–5.00) in patients without PBUB (*p* = 0.3; Table S4).

Large varices were present in 586 EBLs (95%), and varices with high‐risk stigmata were observed in 417 EBLs (68%), with no differences between patients with PBUB and those without PBUB (68% vs. 72%, *p* = 0.7). Gastric varices (49 cases, 7.8%), hiatal hernia (49 cases, 7.9%), and gastroesophageal reflux disease (GERD; 42 cases, 6.7%) were less frequently documented findings in patients with PBUB and those without PBUB (Table S4).

Proton pump inhibitors (PPIs) were used in association with 471 EBL procedures (75%), and non‐selective beta blockers (NSBB) in 468 EBL procedures (74%). PPI use did not differ between patients with PBUB (31 EBLs, 76%) and without PBUB (440 EBLs, 75%, *p* = 0.8). NSBB use was lower in patients with PBUB (24 EBLs, 59%) than in patients without PBUB (444 EBLs, 75%, *p* = 0.049; Table S4).

Antibiotics were administered in all cases of urgent EBL for acute VB and for identified infections in 203 EBL procedures (33%). Antibiotic administration was significantly higher in patients with PBUB (59%) compared to patients without PBUB (31%, *p* = 0.02). Anticoagulation therapy was used on 107 occasions (17%) across all EBLs, with no significant difference between patients with PBUB (27%) and patients without PBUB (16%, *p* = 0.063). Similarly, anti‐aggregation therapy was documented in 77 cases (12%) of all EBLs, with no significant difference in patients with PBUB (20%) and patients without PBUB (12%, *p* = 0.2; Table S4).

### 3.6 Risks Factors Associated to PBUB After EBL

In univariate analysis (Table 4), risk factors significantly associated with an increased risk of PBUB included urgent EBL (SHR: 3.91, 95% CI: 2.08–7.37, *p* < 0.001), high MELD score (SHR: 1.06, 95% CI: 1.01–1.10, *p* = 0.012), the presence of infection (SHR: 4.42, 95% CI: 1.68–11.61, *p* = 0.003), INR (SHR: 1.85, 95% CI: 1.18–2.88, *p* = 0.012), creatinine levels (SHR: 1.05, 95% CI: 1.03–1.08, *p* < 0.001), and total bilirubin (SHR: 1.03, 95% CI: 1.01–1.06, *p* = 0.018). Treatment with NSBB was found to be protective against PBUB (SHR: 0.47, 95% CI: 0.25–0.87, *p* = 0.017).

In multivariate analysis (Table 4), only two factors demonstrated a statistically significant association with PBUB: creatinine with a SHR of 1.04 (95% CI: 1.01–1.07, *p* = 0.024) per 10 μmol increase, indicating that higher creatinine levels were independently associated with an increased risk of PBUB; and urgent EBL (SHR: 2.78, 95% CI: 1.29–6.00, *p* = 0.009), demonstrating that urgent EBL carried a significantly higher risk of PBUB than prophylactic EBL. Other factors studied did not show significant associations with PBUB.

### 3.7 Management of PBUB After EBL

Thirty‐six patients developed PBUB, requiring combined endoscopic and medical therapy that included prophylactic antibiotics, vasoactive drugs and PPIs (administered in 28 cases, 66.7%). EBL was repeated in 11 cases (30.6%). Additional interventions were as follows: local epinephrine injection in 3 cases (7.3%), hemospray in 4 cases (9.5%), hemoclips in 3 cases (7.3%), transjugular intrahepatic portosystemic shunt (TIPS) in 5 cases (11.9%), oesophageal self‐expandable metallic stents (SEMS) in 5 cases (12.2%), oesophageal balloon tamponade in 2 cases (4.9%), and OLT in 1 case (2.5%).

## 4 Discussion

This study shows that the incidence rate of PBUB was 17.5% of the patients treated, and in 6.8% of the EBL procedures. Urgent EBL carried a higher risk of PBUB (SHR: 2.78, *p* = 0.009) as well as elevated creatinine levels (SHR: 1.04, *p* = 0.024). Patients with PBUB required intensive care hospitalisation in 74.4% of cases and had a 19% in‐hospital mortality rate.

The incidence rate of PBUB in our study was higher than those reported in previous studies [10, 11, 12, 13, 14, 15, 16, 17, 18, 19, 20, 21, 22, 23, 24, 25, 26]. A systematic review with meta‐analysis [8] estimated the pooled incidence of PBUB in patients with cirrhosis at 5.5% (95% CI, 4.3–7.1). Among the studies included in the meta‐analysis, the highest reported incidence rates were observed in Dueñas et al. [19] (2020) at 12% and Rohita et al. [16] (2020) at 11.9%. Three factors may explain the higher incidence of PBUB in our cohort: the greater cumulative exposure due to more EBL sessions despite a lower PBUB risk per EBL procedure (6.8%); a higher proportion of urgent EBL with known higher PBUB rates [12, 14, 16, 17, 20, 23, 27]; and possibly unexamined factors such as higher portal pressure, differences in patient selection criteria, and PBUB definition. Vanbiervliet et al. [17] included only post‐slippage ulcers, which represent a subset of cases.

Patients with PBUB exhibited a higher frequency of liver cirrhosis decompensation (69%), including acute VB, hepatic encephalopathy, ascites, jaundice and acute kidney injury, than patients without PBUB (27%), with many requiring urgent EBL and hence increased PBUB risk. Urgent EBL was independently associated with an increased risk of PBUB in our cohort (SHR: 2.78, 95% CI: 1.29–6.00, *p* = 0.009), as previously reported in the literature [14, 23, 25, 27].

MELD score was associated with PBUB in univariate analysis (HR: 1.06, 95% CI: 1.01–1.10, *p* = 0.012) but was not included in the multivariate model to avoid collinearity, as its components—creatinine, INR and total bilirubin—were analysed independently. Only creatinine emerged as a significant risk factor for PBUB, highlighting the role of renal dysfunction in the development of PBUB that may have been masked in studies evaluating MELD [11, 12, 19, 23, 27], as a composite score.

The presence of infection was positively associated with PBUB risk in univariate analysis, but this association was not independent in multivariate analysis. This may suggest that infection acts as a precipitant of decompensation or worsening of liver disease severity, rather than a direct cause of PBUB.

The use of NSBB, PPIs, anticoagulants, or anti‐aggregation therapy was not associated with PBUB. This contrasts with a meta‐analysis [28] suggesting a protective role of PPIs in post‐banding ulcer healing and rebleeding prevention. The low prevalence of anticoagulants and anti‐aggregation use in our cohort may have limited the statistical power.

Post‐banding ulcers (PBU) are a common consequence of EBL (37.9% per patient, 19% per EBL), but only a subset progressed to PBUB, indicating that additional factors, such as urgent EBL and renal impairment, play a more direct role in PBUB risk. There is no universally validated classification for PBUB, and this lack of standardisation *complicates* comparisons across studies. A further limitation for risk‐factor inference is that urgent procedures may carry a higher risk due to technical challenges (limited visualisation, incomplete ligation, greater mucosal trauma during active bleeding), which we could not characterise in this retrospective dataset.

Contemporary guidance recommends repeating EBL every 2–4 weeks until eradication [29]. A randomised trial [30] found that 1‐week intervals shortened the time to eradication but did not improve clinical outcomes compared with 2‐week schedules. In our cohort, inter‐session intervals were similar in patients with and without PBUB.

In patients experiencing PBUB, short‐term mortality during hospitalisation was 19%, with an important decrease in survival over time, especially in the setting of urgent EBL. These findings are consistent with the literature, where a pooled mortality rate of 22.3% was reported in a systematic review and meta‐analysis evaluating 14 articles [8]. Even in a subgroup analysis of patients with prophylactic EBL minimising confounding from urgent procedures for acute VB in decompensated patients, PBUB predicted worse 28–365‐day survival. Residual confounding by urgent EBL for acute VB may exist due to the small numbers of PBUB cases in this subgroup.

The management strategies for PBUB observed in our study are consistent with those reported in the literature [10, 12, 21, 23], even though a true standard is lacking. A stepwise escalation of therapy, from medical to endoscopic and advanced interventions extrapolated from VB or ulcer bleeding, remains the standard approach to PBUB management in our centre.

Relevant strengths of this study include detailed patient‐level data from 630 EBL procedures and long‐term follow‐up. A robust prediction model and preventive approaches would help to optimise outcomes and reduce mortality. For high‐risk patients with Child‐Turcotte‐Pugh B and C, pre‐emptive TIPS placement instead of EBL should be considered in selected cases. TIPS as secondary prophylaxis after EBL and medical treatment may also be an option for patients with signs of persistent portal hypertension or recurrent bleeding. Furthermore, the temporary interruption of NSBB therapy in decompensated patients should be carefully evaluated to ensure that the benefits of portal pressure reduction are not prematurely lost. Future multi‐centre studies exploring the role of portal pressure as a predictor of PBUB may offer valuable insights into the pathophysiology and refine risk assessment.

## 5 Conclusion

Urgent EBL and elevated creatinine levels are independent risk factors for PBUB, particularly in patients with decompensated cirrhosis. PBUB significantly impacts mortality in patients with cirrhosis, emphasising the critical need for improved risk identification and stratification. Developing a robust prediction model to identify high‐risk patients, exploring preventive strategies, and optimising management approaches based on the severity of PBUB are essential steps to reduce its burden and improve patient outcomes.

## Author Contributions

**Maria de Brito Nunes:** investigation, writing – original draft, data curation. **Matthias Knecht:** investigation, writing – review and editing. **Jonas Schropp:** methodology, writing – review and editing, formal analysis. **Reiner Wiest:** investigation, writing – review and editing, resources. **Jaume Bosch:** writing – review and editing, conceptualization. **Annalisa Berzigotti:** conceptualization, writing – review and editing, supervision, resources.

## Funding

This work was supported by the foundation Burgergemeinde Bern (Switzerland).

## Conflicts of Interest

Jaume Bosch is a consultant for Astra‐Zeneca, Boehringer‐Ingelheim, NovoNordisk and Resolution Therapeutics. Annalisa Berzigotti has been a consultant to Boehringer‐Ingelheim. The remaining authors disclose no conflicts.

## Supporting information


**Data S1:** apt70495‐sup‐0001‐Supinfo.docx.

## Data Availability

All data relevant to the study is included in the article or uploaded as Supporting Information S1.
