# Supplementary material for: Post‐Banding Ulcer Bleeding After Endoscopic Ligation: Incidence, Risk Factors and Outcomes in Patients With Cirrhosis
Source: Aliment Pharmacol Ther. 2025 Dec 10;63(8):1099–108. doi: 10.1111/apt.70495 (PMC13021296; doi:10.1111/apt.70495)
Supplement: Supplementary file 1 — Data S1: apt70495‐sup‐0001‐Supinfo.docx. [file APT-63-1099-s001.docx]

**SUPPLEMENTARY FILE**

**Supplementary Table 1:** Variables collected during the study period

| **Collected data** | **Description** |
| --- | --- |
| **Demographic** | Sex and age |
| **Comorbidities and cirrhosis characteristics** | Etiology of cirrhosis, severity Scores of liver disease (Child-Pugh score, Child-Pugh score and the Model for End-Stage Liver Disease (MELD) score) ¸ episodes of hepatic decompensation (ascites, hepatic encephalopathy, variceal bleeding), advanced Chronic Liver Disease (ACLD) |
| **Medication** | Ongoing treatments involving Non-Selective Beta-Blockers (NSBB), proton pump inhibitors, vasoactive drugs, statins, antibiotics, diuretics, antiaggregant agents, and anticoagulants |
| **Laboratory** | Blood count, renal parameters, liver parameters (levels of albumin, aspartate aminotransferase (AST), alanine aminotransferase (ALT), and bilirubin), coagulation parameters (prothrombin time (PT), partial thromboplastin time (PTT), and fibrinogen), infectious parameters (leukocyte counts, neutrophil-to-lymphocyte ratio (N/L ratio), and C-reactive protein (PCR) levels |
| **Endoscopic procedures** | Setting of EBL (primary prophylaxis, secondary prophylaxis, acute bleeding), grade, site of treated varices, number of bands placed per endoscopy and also per varix, presence of high-risk stigmata (red wale sign, nipple sign, cherry red spots, fibrin plug) presence of hiatal hernia and assessment using the Hill classification, endoscopic evidence of oesophageal reflux, experience of the endoscopist. |
| **EBL complications and management** | Variceal re-bleeding, PBU, PBUB, interval between ligation and bleeding, severity of PBUB: admission at the intensive care unit (ICU), ongoing infections as aspiration pneumonia, transfusions, need of other life-supporting therapies, transplantation and mortality |

**Supplementary table 2**: Baseline characteristics of overall enrolled patients, patients with PBUB and patients without PBUB during the study observation. Some patients have more than one cirrhosis etiology, and manifestations of the decompensation.

| **Characteristic** | **Overall**  N = 206^1^ | **No PBUB**  N = 170^1^ | **PBUB**  N = 36^1^ | **p-value**^2^ |
| --- | --- | --- | --- | --- |
| **Sex = male, n (%)** | 156 (76%) | 129 (76%) | 27 (75%) | >0.9 |
| **Age (years), median [Q1, Q3]** | 62 [54, 70] | 63 [54, 70] | 61 [52, 70] | 0.6 |
| **Cirrhosis etiology** |  |  |  |  |
| **- Alcohol-associated liver disease (ALD), n (%)** | 126 (61%) | 105 (62%) | 21 (58%) | 0.7 |
| **- Metabolic dysfunction-associated steatotic liver disease (MASLD), n (%)** | 60 (29%) | 47 (28%) | 13 (36%) | 0.3 |
| **- Viral hepatitis (HBV, HCV), n (%)** | 37 (18%) | 32 (19%) | 5 (14%) | 0.5 |
| **- Autoimmune hepatitis, n (%)** | 6 (2.9%) | 6 (3.5%) | 0 (0%) | 0.6 |
| **- Cholestatic disease (PBC, PSC, SSC), n (%)** | 10 (4.9%) | 7 (4.1%) | 3 (8.3%) | 0.4 |
| **- Other, n (%)** | 19 (9.2%) | 18 (11%) | 1 (2.8%) | 0.2 |
| **MELD Score, median [Q1, Q3]** | 12 [9, 18] | 12 [9, 17] | 14 [9, 19] | 0.5 |
| **Child-Pugh Score, median [Q1, Q3]** | 8.00 [6.00, 9.00] | 8.00 [6.00, 9.00] | 8.00 [7.00, 9.00] | 0.5 |
| **Etiology of decompensation** |  |  |  |  |
| **Infection** | 14 (6.8%) | 11 (6.5%) | 3 (8.3%) | 0.7 |
| **Alcohol liver injury** | 57 (28%) | 46 (27%) | 11 (31%) | 0.7 |
| **Treatment withdrawal (diuretics, lactulose, B-blockers)** | 2 (1.0%) | 2 (1.2%) | 0 (0%) | >0.9 |
| **New diagnosis of Portal vein thrombosis** | 10 (4.9%) | 7 (4.1%) | 3 (8.3%) | 0.4 |
| **New diagnosis of HCC or HCC progression** | 17 (8.3%) | 15 (8.8%) | 2 (5.6%) | 0.7 |
| **Other/unclear** | 30 (15%) | 25 (15%) | 5 (14%) | 0.9 |
| **Manifestation of decompensation** |  |  |  |  |
| **- Upper gastrointestinal bleeding, n (%)** | 131 (64%) | 106 (62%) | 25 (69%) | 0.4 |
| **- Ascites, n (%)** | 60 (29%) | 47 (28%) | 13 (36%) | 0.3 |
| **- Hepatic encephalopathy, n (%)** | 39 (19%) | 31 (18%) | 8 (22%) | 0.6 |
| **- Jaundice (new onset), n (%)** | 8 (3.9%) | 6 (3.5%) | 2 (5.6%) | 0.6 |
| **- Respiratory failure, n (%)** | 1 (0.5%) | 1 (0.6%) | 0 (0%) | >0.9 |
| **- Acute kidney failure, n (%)** | 27 (13%) | 18 (11%) | 9 (25%) | 0.029 |
| **- Infection, n (%)** | 4 (1.9%) | 3 (1.8%) | 1 (2.8%) | 0.5 |
| **- Other, n (%)** | 1 (0.5%) | 1 (0.6%) | 0 (0%) | >0.9 |
| **Portal vein thrombosis, n (%)** | 38 (23%) | 29 (22%) | 9 (28%) | 0.5 |
| **Hepatocellular carcinoma, HCC n (%)** |  |  |  | 0.2 |
| **no HCC present** | 150 (73%) | 128 (75%) | 22 (61%) |  |
| **single tumor < 2cm** | 11 (5.3%) | 7 (4.1%) | 4 (11%) |  |
| **single, or up to 3 nodules each up to 3cm** | 15 (7.3%) | 11 (6.5%) | 4 (11%) |  |
| **multinodular** | 18 (8.7%) | 14 (8.2%) | 4 (11%) |  |
| **infiltrative, metastases** | 12 (5.8%) | 10 (5.9%) | 2 (5.6%) |  |
| ^1^n (%); Median [Q1, Q3] | | | | |
| ^2^Pearson's Chi-squared test; Wilcoxon rank sum test; Fisher's exact test | | | | |
|  | | | | |

**Supplementary Table 3. Rebleeding outcomes by gastro-oesophageal varices (GOV) in the full cohort (n=206).**

| **Characteristic** | **no gastro-oesophageal varices**  N = 186^1^ | **gastro-oesophageal varices**  N = 20^1^ | **p-value**^2^ |
| --- | --- | --- | --- |
| Early Variceal rebleeding (<6 weeks) | 72 (39%) | 9 (45%) | 0.6 |
| Late Variceal rebleeding  (>6 weeks) | 54 (29%) | 4 (20%) | 0.4 |
| Any PBUB during observation | 34 (18%) | 2 (10%) | 0.5 |
| ^1^n (%) | | | |
| ^2^Pearson's Chi-squared test; Fisher's exact test | | | |

Early (≤6 weeks) and late (≥6 weeks) variceal rebleeding are defined per Baveno for patients with an index acute variceal bleed; for patients without an index acute bleed (prophylactic setting), timing is referenced to the first EBL session and should be interpreted descriptively.

Supplementary table 4: Therapies and endoscopic findings.

| **Characteristic** | **Overall**  N = 630^1^ | **No PBUB**   N = 589^1^ | **PBUB**   N = 41^1^ | **p-value**^2^ |
| --- | --- | --- | --- | --- |
| **Proton pump inhibitors (PPIs)** | 471 (75%) | 440 (75%) | 31 (76%) | 0.8 |
| **Antibiotic therapy** | 208 (33%) | 184 (31%) | 24 (59%) | 0.002 |
| **Anticoagulation therapy** | 107 (17%) | 96 (16%) | 11 (27%) | 0.063 |
| **Antiaggregant therapy** | 77 (12%) | 69 (12%) | 8 (20%) | 0.2 |
| **Non-selective beta-blockers (NSBB)** | 468 (74%) | 444 (75%) | 24 (59%) | 0.049 |
| **EBL indication** |  |  |  | <0.001 |
| **Acute Variceal Bleeding** | 191 (30%) | 166 (28%) | 25 (61%) |  |
| **Primary Prophylaxis** | 136 (22%) | 130 (22%) | 6 (15%) |  |
| **Secondary Prophylaxis** | 303 (48%) | 293 (50%) | 10 (24%) |  |
| **Presence of gastric varices** | 49 (7.8%) | 47 (8.0%) | 2 (4.9%) | 0.7 |
| **Large varices** | 586 (95%) | 547 (95%) | 39 (100%) | 0.038 |
| **Varices with high-risk stigmata** | 417 (68%) | 389 (68%) | 28 (72%) | 0.7 |
| **Presence of hiatal hernia** | 49 (7.9%) | 44 (7.6%) | 5 (12%) | 0.8 |
| **GERD** | 42 (6.7%) | 38 (6.5%) | 4 (9.8%) | 0.4 |
| **Number of bands placed per endoscopy** | 3.00 [2.00, 4.00] | 3.00 [2.00, 4.00] | 3.00 [2.00, 4.00] | 0.6 |
| **Number of bands at first EBL** | 3.00 [2.00, 5.00] | 3.00 [2.00, 5.00] | 4.00 [2.50, 5.00] | 0.3 |
| **Type of PBUB** |  |  |  | <0.001 |
| **Type A** | 7 (6.8%) | 0 (0%) | 7 (17%) |  |
| **Type B** | 2 (1.9%) | 1 (1.6%) | 1 (2.4%) |  |
| **Type C** | 28 (27%) | 5 (8.1%) | 23 (56%) |  |
| **Type D** | 66 (64%) | 56 (90%) | 10 (24%) |  |
| ^1^Median [Q1, Q3]; n (%) | | | | |
| ^2^random intercept logistic regression | | | | |

**Supplementary table 5**: Laboratory findings at the time of EBL of overall enrolled patients, patients with PBUB and patients without PBUB during the study observation

| **Characteristic** | **Overall**  N = 206^1^ | **No PBUB**   N = 170^1^ | **PBUB**  N = 36^1^ | **p-value**^2^ |
| --- | --- | --- | --- | --- |
| **Hemoglobine (g/L)** | 92 [76, 112] | 93 [76, 111] | 90 [76, 114] | 0.7 |
| **Platelets(G/L)** | 97 [70, 137] | 94 [71, 137] | 108 [58, 141] | 0.9 |
| **Leukocytes (G/L)** | 5.8 [4.3, 9.4] | 5.8 [4.2, 9.4] | 6.5 [4.5, 10.4] | 0.5 |
| **Neutrophiles(G/L)** | 3.7 [2.5, 6.8] | 3.8 [2.6, 6.1] | 3.4 [2.1, 7.2] | 0.9 |
| **Lymphocyte(G/L)** | 1.14 [0.78, 1.61] | 1.14 [0.80, 1.47] | 1.12 [0.78, 1.63] | 0.9 |
| **PCR/CRP(mg/L)** | 13 [5, 25] | 11 [4, 25] | 18 [6, 30] | 0.13 |
| **Glucose(mmol/L)** | 6.48 [5.39, 8.00] | 6.56 [5.39, 8.10] | 6.00 [5.25, 8.00] | 0.6 |
| **Albumin(g/L)** | 29 [23, 32] | 29 [23, 32] | 31 [25, 33] | 0.3 |
| **Aspartate aminotransferase-AST (U/L)** | 56 [39, 91] | 54 [38, 81] | 64 [41, 110] | 0.2 |
| **Alanine aminotransferase-ALT (U/L)** | 35 [25, 52] | 34 [25, 53] | 38 [25, 52] | 0.5 |
| **Gamma-glutamyltransferase-GGT (U/L)** | 139 [66, 263] | 138 [66, 259] | 145 [90, 349] | 0.5 |
| **Alkaline phosphatase(U/L)** | 123 [93, 172] | 121 [87, 175] | 131 [102, 161] | 0.4 |
| **Total bilirubin(umol/L)** | 27 [14, 48] | 26 [14, 47] | 28 [13, 59] | 0.6 |
| **Sodium (mmol/l)** | 138.0 [134.0, 140.0] | 138.0 [134.0, 140.0] | 137.5 [134.0, 140.0] | 0.5 |
| **Potassium (mmol/l)** | 4.10 [3.80, 4.60] | 4.10 [3.70, 4.60] | 4.10 [3.80, 4.40] | >0.9 |
| **Creatinine(umol/L)** | 80 [62, 115] | 77 [61, 113] | 91 [73, 144] | 0.023 |
| **Urea (mmol/L)** | 7 [5, 13] | 7 [5, 12] | 8 [5, 16] | 0.4 |
| **Alpha-fetoprotein (KU/L)** | 3 [2, 6] | 3 [2, 6] | 5 [2, 7] | 0.4 |
| **Unknown** | 98 | 80 | 18 |  |
| **INR** | 1.27 [1.16, 1.53] | 1.27 [1.16, 1.52] | 1.28 [1.17, 1.58] | 0.9 |
| **Prothrombin time (%)** | 60 [44, 71] | 60 [45, 71] | 59 [43, 70] | 0.6 |
| **PPT (sec)** | 40 [33, 57] | 39 [33, 54] | 52 [37, 67] | 0.090 |
| **Unknown** | 117 | 97 | 20 |  |
| **Fibrinogen(g/L)** | 1.86 [1.37, 2.57] | 1.94 [1.45, 2.50] | 1.52 [1.17, 2.74] | 0.4 |
| ^1^Median [Q1, Q3] | | | | |
| ^2^Wilcoxon rank sum test | | | | |

**Supplementary Figure 1 :Violin plot with median/IQR box and individual patients.**


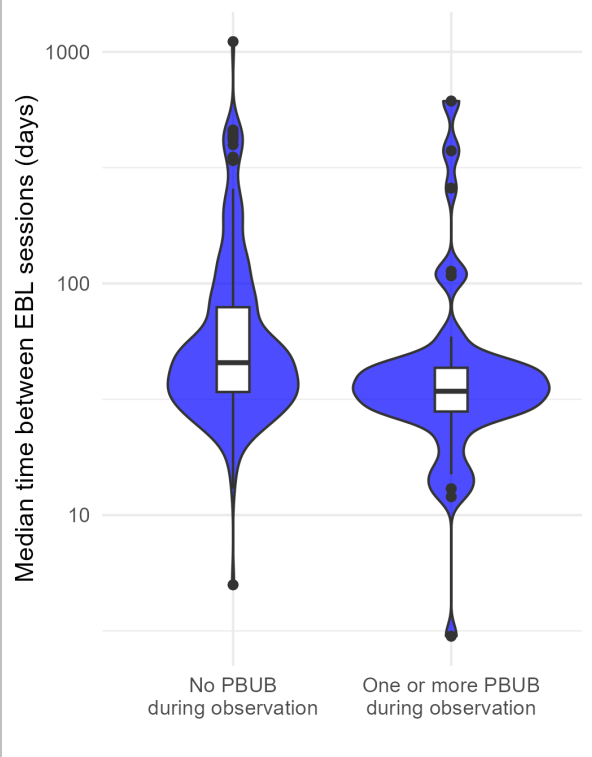


y-axis = days (log scale).
